# Method for emissivity measurement of semitransparent coatings at ambient temperature

**DOI:** 10.1038/s41598-017-01574-x

**Published:** 2017-05-03

**Authors:** Petra Honnerová, Jiří Martan, Zdeněk Veselý, Milan Honner

**Affiliations:** New Technologies Research Centre (NTC), University of West Bohemia, Univerzitní 8, 306 14 Pilsen, Czech Republic

## Abstract

Coatings deposited on a material surface are effective way of changing its surface properties. For increasing or decreasing radiation heat transfer, coatings with high or low emissivity are used. Measurement of spectral emissivity is a fundamental step to effective use of coatings for this application. Up to now the measurement methods are focused on bulk samples and mainly opaque ones. Here we present a method enabling measurement of emissivity of semitransparent coating itself, although it is deposited on a substrate. The method is based on measurement of transmittance and reflectance using an integration sphere system and Fourier transform infrared (FTIR) spectrometer for samples with two different coating thicknesses deposited on transparent substrates. Measured transmittance of the coating indicates spectral regions of potential emissivity differences using different substrates. From all the measured values, spectral emissivity can be characterized for different coating thicknesses. The spectral range of the method is from 2 μm to 20 μm. The measurement is done at ambient temperature enabling measurement of samples sensitive to heating like biomedical or nanocoatings. The method was validated on known bulk samples and an example of semitransparent coating measurement is shown on high-temperature high-emissivity coating.

## Introduction

The emissivity is a material property that characterizes the ability of a real surface to emit radiation. It is the basic property describing radiation heat transfer. By definition, the emissivity of a body is a ratio of radiances of its surface and ideal black body both at the same temperature and identical spectral and geometrical conditions. The emissivity is dependent on the material, surface properties, surface state, surface temperature, wavelength, direction of the radiation and polarization grade. The values range from 0 to 1. Directional spectral emissivity is a basic property that can be used for the evaluation of other properties of emissivity, for example, effective emissivity or total emissivity.

Direct or indirect methods are generally used to the determination of directional spectral emissivity of bulk materials. The direct methods^1, 2^ compare the radiations from a sample and a blackbody. The radiations are measured by radiation thermometer or spectrometer especially at higher temperatures. For ambient temperatures, the indirect methods are preferred because of higher radiation intensity below wavelength of 3 μm^2^. The methods are based on the measurement of spectral reflectance *ρ*_*λ*_ (*λ*, *T*) and transmittance *τ*_*λ*_ (*λ*, *T*). The spectral emissivity *ε*_*λ*_ (*λ*, *T*) can be evaluated according to Kirchhoff’s law^3^

1 $${\varepsilon }_{\lambda }(\lambda ,T)={\alpha }_{\lambda }(\lambda ,T)=1-{\rho }_{\lambda }(\lambda ,T)-{\tau }_{\lambda }(\lambda ,T),$$

where *α*_*λ*_ (*λ*, *T*) is spectral absorptance, *λ* is wavelength and *T* is temperature.

The integrating sphere systems with spectrometers are suitable devices for the measurement of spectral reflectance and transmittance of different kinds of samples. According to the integrating sphere system constructions, the spectral quantities are measured in a relative mode^4–7^ (against standard material with known properties) or in an absolute mode^4, 8^ (independent of a reference), both in a direct^9–12^ or an indirect^9, 13, 14^ conditions. In the direct conditions, the radiation from a radiation source is incident the first on the sample. In the other case, the radiation is directed to the inner surface of integrating sphere, where it is reflected and the measured sample is subsequently diffusely illuminated.

At the New Technologies Research Centre (NTC) of the University of West Bohemia in Pilsen, the direct radiometric method for spectral emissivity measurement of coatings at high temperatures (300 °C–1000 °C) has been developed in last years^15^. The method enables to characterize the behavior of coatings deposited on the ceramic or metal substrates in the spectral range from 1.38 μm to 26 μm. In the case of semitransparent coatings, the emissivity of the coating/substrate system is determined. The coatings are used to influence the efficiency of heat transfer in the high-temperature applications^16–18^ or in the area of ambient temperatures, for example in photovoltaics/solar^19, 20^, buildings^7, 21, 22^, military^23^ or microelectromechanical systems^24, 25^. Therefore, the method of emissivity measurement of coatings at ambient temperatures has been developed as a supplement to the direct radiometric high-temperature emissivity measurement method. This method could be developed also for elevated temperatures (up to 300 °C) in future, but would need homogeneous sample heating and integration sphere cooling.

Many of the mentioned coatings for ambient temperature applications are composed on layers or composite structures with nanometer scale (nanocoatings). Some of them are also deposited on polymer substrates. These materials are usually sensitive to heating (temperature increase). The presented method does not require heating of the sample for measurement of emissivity and thus it is safe for the temperature sensitive samples. Measurement of emissivity of semitransparent coatings on semitransparent substrates is not an easy task and is not routinely performed in optical laboratories. Up to now the measurement methods are focused on bulk samples and mainly opaque ones. The coating with thickness in nanometers and micrometers cannot be removed from the substrates for stand-alone emissivity measurement. Here we present a method enabling measurement of emissivity of semitransparent coating itself, although it is deposited on a substrate. This is very important for the coatings development and application.

The Fourier transform infrared (FTIR) spectrometer was extended by a commercial integrating sphere system operating in the IR spectral range. Usually, the relative normal hemispherical reflectance of bulk samples and the normal hemispherical transmittance of thin sheets can be measured. At the NTC, two arrangements of the commercial system have been done. The first, an accessory for normal hemispherical transmittance measurement of bulk samples (or coating/substrate systems) has been developed. The second, a suitable standard reference material with known properties was selected. Thus, the absolute reflectance and transmittance of bulk materials can be evaluated. Furthermore, measurement and evaluation process has been also developed and optimized and emissivity of semitransparent coatings deposited on the bulk transparent substrate can thus be calculated.

In this paper, the specifics of integrating sphere system including accessory for normal hemispherical transmittance measurement of bulk samples is described in Experimental Device section. The selection of standard reference material and the measurement process optimization are discussed in Measurement Process Optimization section. The final measurement procedure, considered sources of uncertainty and their evaluation are commented in Measurement and Evaluation Procedure section and Measurement Uncertainty section. Examples of method application are presented in Experimental results section.

## Experimental Device

**Figure 1:**
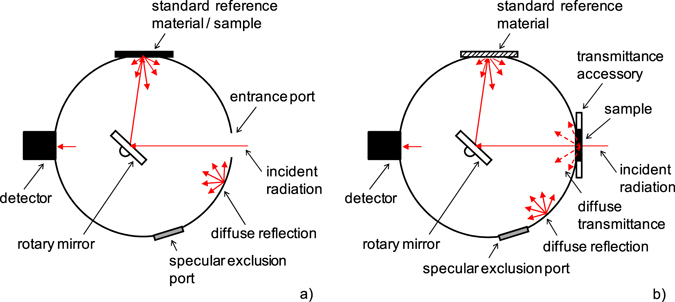
Schematic view of integrating sphere system. (**a**) Normal hemispherical reflectance measurement mode. (**b**) Normal hemispherical transmittance measurement mode.

The reflected or transmitted radiation by the sample is measured using a FTIR spectrometer to which is attached an integrating sphere system. The FTIR spectrometer Nicolet 6700 and commercial integrating sphere system IntegratIR^TM^ from PIKE Technologies^26^ are used. The sphere diameter is 76.2 mm and its inner surface is coated by a high reflectance diffuse gold coating. The entrance port with a diameter of 24.5 mm is situated on side of the sphere, the reflectance sample port with a diameter 20 mm is positioned on top of the sphere. A Hg:Cd:Te detector records the reflected or transmitted radiation by the sample in the spectral range from 2 μm to 20 μm. The measurement of proportion of specular and diffuse components of transmittance and reflectance of analyzed samples can be performed as well. The direct measurement conditions^10, 11^ are respected. The schematic view of integrating sphere design is shown in Fig. 1.

In the reflectance measurement, the radiation from an internal source of the spectrometer enters into the integrating sphere through the entrance port and is reflected by the rotary mirror to the sample port. A standard reference material or a sample placed on the reflectance sample port is irradiated under the angle of 12°. The radiation reflected by the standard reference material or the sample is repeatedly reflected inside the integrating sphere until it leaks through the detector port onto the detector. In the transmittance mode, the radiation from the internal source of the spectrometer passes through the sample placed in front of the entrance port. Subsequently, the radiation enters the integrating sphere system, where it is repeatedly reflected until it leaks through the detector port onto the detector. The reflectance sample port is enclosed by the standard reference material.

**Figure 2:**
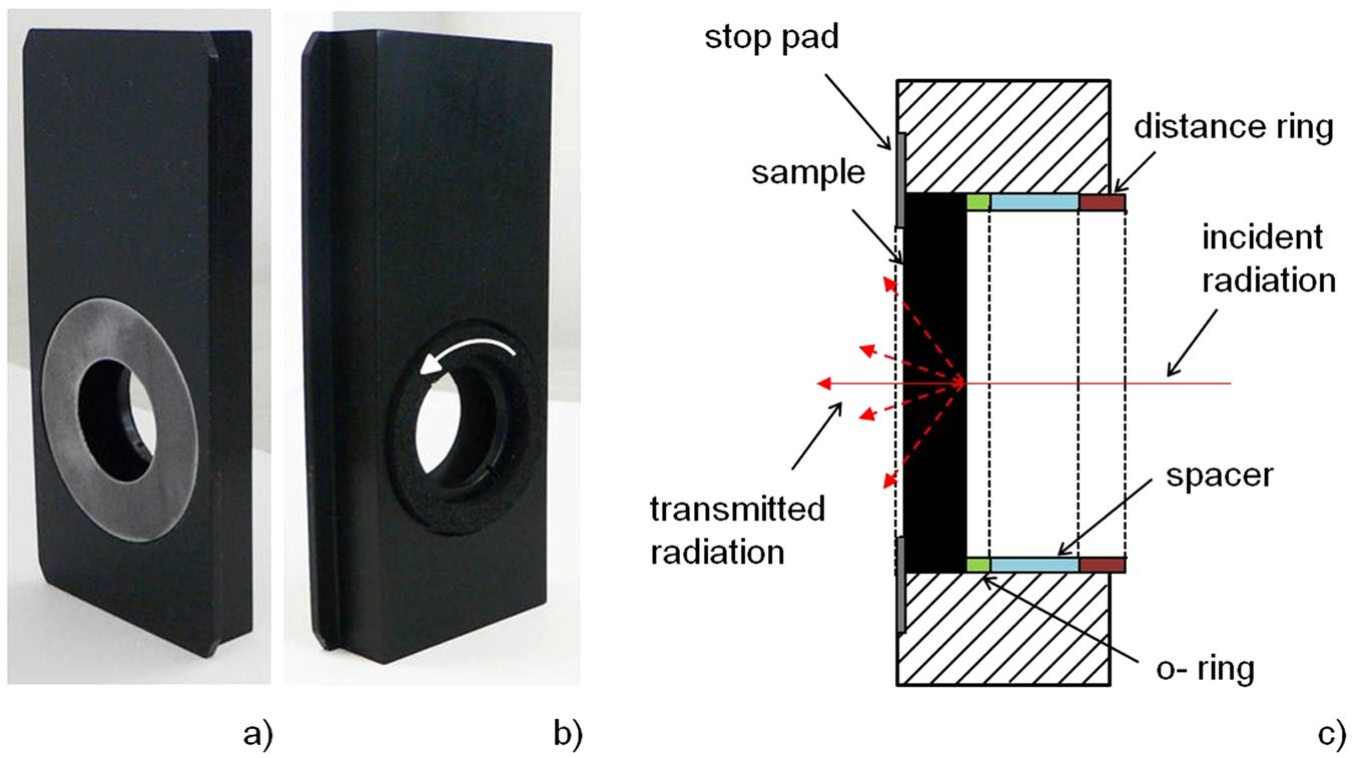
Transmittance accessory for bulk samples measurement. (**a**) Photograph of front view. (**b**) Photograph of back view. (**c**) Detail schematic view of sample port.

Transmittance measurement of thin sheets only is enabled by the commercial integrated sphere system. Therefore, two accessories have been developed for bulk samples measurement (see Fig. 2). The both accessories are designed on the same principle, the measurement of various samples (shapes and sizes) is allowed. The analyzed sample is inserted into the accessory sample port up to a stop pad and locked into the measurement position by using the spacer and distance ring. An o-ring is placed between the sample and spacer to avoid the sample damage. The different thicknesses of spacers are used to maintain the exact position of distance ring when different thicknesses of samples are measured. The distance ring fixes all the components of accessory together and avoids leakage of radiation from optical path.

The spectral normal hemispherical transmittance and reflectance of the bulk samples of circular shape with diameter from 24 mm to 38 mm or square with a side length 25.5 ± 1 mm can be measured. The sample thickness (coating/substrate system) is in the range from 0.01 to 7 mm. This is due to the experimental configuration, the construction of apparatus and transmittance accessory. The thickness of coating itself can be from nanometers to hundreds of micrometers. According to Kirchhoff’s law (equation (1)), the bulk samples emissivity can be calculated. Similarly, the optical properties of coatings deposited on the bulk transparent substrates are evaluated.

## Measurement Process Optimization

### Standard reference materials

It is supposed to measure samples with a wide range of reflectance and surface conditions using the integrating sphere system. As recommended^27^, various standard materials should be used for the various samples. In total, five candidates of materials were chosen as a suitable standard reference material for reflectance measurement (directional hemispherical reflectance standard – DHR standard) with respect to their physical properties:

- uncoated molybdenum laser mirror with diameter of 76.2 mm (MoStan) with reflectance higher than 0.95, specular standard material, company Laser Beam Products Ltd, UK, calibrated in Laboratoire National d’Essais (LNE) in France;
- Germanium standard material with reflection about 0.36 from the front surface only (GeStan), specular standard material, company Middleton Spectral Vision, USA;
- Avian Gold Diffuse Standard with reflectance about 0.93 (AuStan), diffuse standard material, company Avian Technologies LLC, USA;
- Decrylate-Coated Flame Sprayed Aluminium with reflectance about 0.43 (DCAStan), diffuse standard material, National Physical Laboratory, UK;
- Nextel Black on Aluminium with reflectance about 0.03 (NBStan), diffuse standard material, National Physical Laboratory, UK.

The DHR standards vary in the surface conditions (specular or diffuse) and reflectance values. The property requirements^27^, such as opacity, planarity, portability, inertness or spectral flatness are observed.

The standards were calibrated in different national laboratories at angles close to the surface normal (less than 10° to the surface normal). The specular standards were calibrated without polarization. If one of the above standards is used, the absolute spectral normal hemispherical reflectance of measured sample can be evaluated.

The surface uniformity and azimuth angle uniformity of DHR standards were analyzed in our laboratory. Furthermore, the use of standards for samples with different properties was tested and one DHR standard was evaluated as suitable for the analysis of specular and diffuse materials and materials with varying degrees of reflectance.

**Figure 3:**
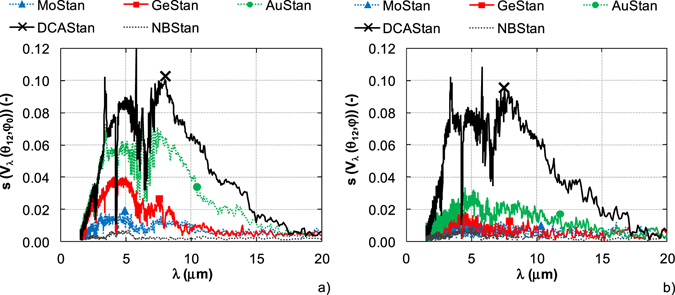
Uniformity of DHR standards. (**a**) Surface uniformity. (**b**) Azimuth angle uniformity.

At first, the surface uniformity of DHR standards was analyzed. In total, nine spectral signals were collected for each DHR standard. The center position of standard on the standard reference port of integrating sphere system was chosen as the initial position. The other positions correspond to the deviation from the initial position of ±1 mm and ±1 cm in the direction of x and y axes. The surface uniformity of DHR standards was evaluated as the standard deviation of measured spectral signals *V*_*λ*_ (*θ*_*12*_, *φ*_*0*_), where *θ* is incidence angle and *φ* is azimuth angle. The results are shown in Fig. 3a.

The azimuth angle uniformity was evaluated similarly as the surface uniformity. The initial position of standard on the standard reference port of integrating sphere system corresponded to the center position with azimuth angle *φ*  = 0°. The other positions *φ* = 45°, 90° and 135° relative to the initial position were used. In total, four spectral signals *V*_*λ*_ (*θ*_*12*_, *φ*) were measured and the standard deviation was calculated. The results are shown in Fig. 3b.

In the both cases, the best surface uniformity and azimuth angle uniformity was achieved for diffuse material with reflectance about 0.03 (NBStan) and specular material with reflectance higher than 0.95 (MoStan). The specular standard with reflectance about 0.36 (GeStan) also indicates the high azimuth angle uniformity. On the contrary, the diffuse standard with middle reflectance (DCAStan) has the worst surface uniformity and azimuth angle uniformity. Therefore, the most suitable DHR standards in terms of uniformity are MoStand and NBStan. In our test it is assumed an ideal behavior of the sphere coating and an ideal sphere geometry.

**Figure 4:**
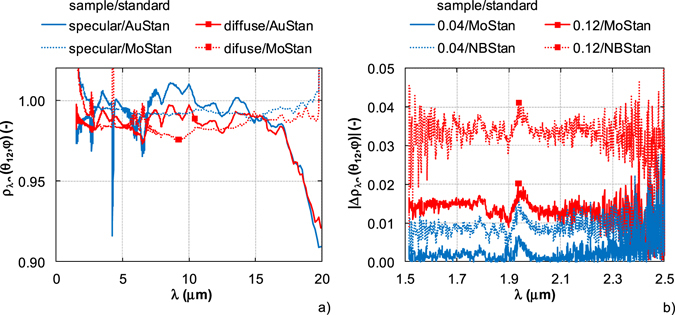
Selection of DHR standards according to characteristics of analyzed samples. (**a**) Specular vs. diffuse surfaces. (**b**) Low reflectance surfaces – reflectance differences.

The operative use of DHR standards also depends on the properties of analyzed samples. The matte or glossy surfaces of DHR standards are required for different applications, the high reflectance is recommended in many cases^27^. The specular and diffuse samples with different reflectance were tested against the specular and diffuse DHR standards. The reflectance of specular and diffuse gold films with high reflectance was measured with specular standard MoStan and diffuse standard AuStan and absolute reflectance *ρ*_*λ*∩_ (*θ*_*12*_, *φ*) was evaluated (Fig. 4a). Similarly, the absolute reflectance of two calibrated samples with known low reflectance (0.04 and 0.12) was calculated from the relative reflectance measured against the high reflectance standard MoStand and low reflectance standard NBStan. The samples were calibrated only in the spectral range from 0.2 μm to 2.5 μm. Absolute values of differences |Δ*ρ*_*λ*∩_ (*θ*_*12*_, *φ*)| between known and calculated reflectance are shown in Fig. 4b.

The diffuse and specular gold films (Fig. 4a) measured with the specular and diffuse DHR standards have similar reflectance. In the both cases, the reflectance is higher than 0.97 for wavelengths up to 17 μm. The significant differences in the results are evident for wavelengths over 17 μm. While spectral curves measured against the specular standard MoStan are almost constant, the reflectance measured with diffuse standard AuStan sharply decreases. Furthermore, the reflectance of specular gold film measured with diffuse standard is higher than 1 in the spectral band from 7 to 10 μm. On the contrary, the reflectance higher than 1 was evaluated for wavelength lower than 2.2 μm and higher than 19 μm if specular standard MoStan was used. Smoother spectral curves of sample reflectance are achieved with MoStan.

In Fig. 4b, better approximation to the known (calibrated) value of reflectance is obtained for the measurement of the two low reflectance samples with high reflectance standard MoStan. The difference between known and measured sample reflectance is almost negligible when the MoStan is used for the sample with reflectance about 0.04. If the standard with low reflectance is applied, the difference is about 0.01. For the sample with reflectance about 0.12, the difference between known and measured sample reflectance is about 0.015 (MoStan) and 0.03 (NBStan).

To summarize the measurement results, the best results of surface uniformity and azimuth angle uniformity are achieved for high reflectance standard MoStan and low reflectance standard NBStan. More reliable reflectance results of specular and diffuse samples are obtained for specular standard MoStan. Simultaneously, better results are read with high reflectance standard MoStan if low reflectance materials are measured. Therefore, DHR standard MoStan is selected as the universal standard for diffuse and specular materials and for materials with low to high reflectance. The long-term stability of MoStan can be affected by the surface oxidation. Therefore, a periodic standard calibration should be assumed.

For transmittance measurement, no standard reference material is used. The standard reference port of integrating sphere system (Fig. 1b) is covered by the material identical to the sphere wall. So, all transmitted radiation is detected.

### Transmittance accessory

When the transmittance of bulk materials and coatings is measured, the reference spectral signal and the sample spectral signal are detected subsequently. The reference spectral signal corresponds to the radiation from the radiation source that passes through the transmittance accessory without the sample (for bulk samples) or through the transmittance accessory with a transparent substrate (for coatings). The same measurement conditions should be ensured for the reference and sample spectral signal measurement.

The distance ring fixes all the components of transmittance accessory (sample, o-ring and spacer) together (Fig. 2c) and avoids leakage of radiation from optical path. If the reference spectral signal is measured, the distance ring position can be varied by the sample thickness or different spacers thicknesses should be used to ensure the same distance ring position during measurement of reference and sample signal. Therefore, the dependence of spectral signal on the distance ring position was measured.

**Figure 5:**
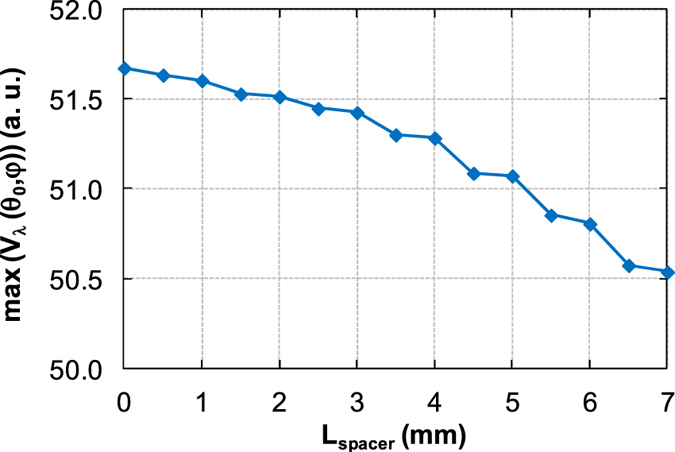
Maximum of spectral signals for different distance ring positions of transmittance accessory.

In total, fifteen spectral signals were measured with the transmittance accessory and the maximum values *max*(*V*_*λ*_ (*θ*_*0*_, *φ*)) were deducted for the different distance ring positions (Fig. 5). The distance ring was positioned behind the stop pad in the default position. For other distance ring positions, the spacers of different thicknesses were inserted between the stop pad and the distance ring. Spacer thicknesses from 0.5 mm to the 7 mm with the step of 0.5 mm were used.

The maximum of spectral signals decreases with the increasing spacer thickness. This means that the measured spectral signal depends on the distance ring position. Therefore, the same position of distance ring should be used for the reference and sample spectral signal measurement to ensure the same measurement conditions. If the distance ring position for the sample signal is set up such that the maximum thickness of sample was simulated, the same distance ring position should be used for the reference signal measurement. The space between the stop pad and the distance ring should be completed by the spacers of adequate thickness for reference and sample spectral signal measurement.

## Measurement and Evaluation Procedure

### Measurement conditions

The apparatus preparation is required before the reflectance and transmittance measurement (Fig. S1). The Hg:Cd:Te detector of integrating sphere system must be cooled by liquid nitrogen and then temperature stabilized. It is appropriate to cool the detector after the connection to the interface of spectrometer and stabilize for 140 minutes. Subsequently, it is possible to begin the measurement. The detector sensitivity decreases after 6.5 hours from temperature stabilization. For farther measurement, the detector should be cooled and stabilized again. Detailed information about the integrating sphere operation are in Integrating Sphere System operation section of supplementary information online.

No evacuating or purging of the measurement apparatus is used. The results are affected by the change of atmospheric absorptions due to varying partial pressures of H_2_O and CO_2_ in spectral ranges 2.5 μm to 2.95 μm, 4.17 μm to 4.5 μm, 4.8 μm to 8 μm and 13.2 μm to 17.2 μm. The back reflected radiation is not included to the reflectance and transmittance measurement (Fig. S2 and Back Reflected Radiation section online).

Each spectrum is measured with the spectral resolution of 8 cm^−1^ and 250 scans of the FTIR spectrometer. The speed of movable mirror of interferometer is 1.26 mm∙s^−1^. The samples are analyzed in the spectral range from 2 μm to 20 μm.

### Transmittance measurement

The following measurement procedure is used for the transmittance measurement. The standard reference port of integrating sphere system is covered by the high reflectance diffuse gold material identical to the sphere inner wall. Generally, one reference signal *V*_*λ ref*_ (*θ*_*0*_, *φ*) and one sample spectral signal *V*_*λ*_ (*θ*_*0*_, *φ*) are collected. The transmittance *τ*_*λ*∩_ (*θ*_*0*_, *φ*) is evaluated as the ratio of the two signals according to

2 $${\tau }_{\lambda \cap }({\theta }_{0},\phi )=\frac{{V}_{\lambda }({\theta }_{0},\phi )}{{V}_{\lambda ref}({\theta }_{0},\phi )}.$$

The ideal sphere geometry is assumed without the sample internal diffusion caused by the multiple internal scattering of incident radiation within the sample^28^.

When the bulk samples without coating are analyzed, the reference spectral signal corresponds to the radiation from the radiation source that passes through the transmittance accessory without the sample. The sample spectral signal corresponds to the radiation passes through the sample.

Transmittance evaluation of semitransparent coatings deposited on the transparent substrates involves substrate transmittance measurement and transmittance measurement of the coating/substrate system. The transmittance of coatings itself is simplified evaluated as the ratio of coating/substrate system transmittance to the substrate transmittance. The evaluation neglects the coupling radiative transfer process within the semitransparent coating and the transparent substrate^29^. When the measurement is provided at room temperature, the contribution of radiative transfer is small comparing to high temperature measurement.

For all measurement, the distance ring position corresponds to the maximum possible thickness of sample. The space between the stop pad and distance ring, resp. between the o-ring and distance ring is filled by the spacers of adequate thickness. The reference spectral signal is measured for each sample.

The analyzed area dimensions depend on the thickness of analyzed sample (Fig. S3a and Analyzed Area Dimensions section online). This varies from 19 mm (for sample thickness 0.01 mm) to 22 mm (for sample thickness 7 mm).

### Reflectance measurement

The reflectance measurement of opaque bulk materials and semitransparent bulk materials is based on the principle similar to the transmittance measurement. The reference signal *V*_*λ ref*_ (*θ*_*12*_, *φ*) and the sample signal *V*_*λ*_ (*θ*_*12*_, *φ*) are detected by the spectrometer. The reference signal is related to the radiation intensity reflected from DHR standard placed on the reflectance port of integrating sphere system. The spectral normal hemispherical reflectance *ρ*_*λ*∩_ (*θ*_*12*_, *φ*) is evaluated according to

3 $${\rho }_{\lambda \cap }({\theta }_{12},\phi )=\frac{{V}_{\lambda }({\theta }_{12},\phi )}{{V}_{\lambda ref}({\theta }_{12},\phi )}\cdot {\rho }_{\lambda ref}({\theta }_{12},\phi )={\rho }_{\lambda \cap ,rel}({\theta }_{12},\phi )\cdot {\rho }_{\lambda ref}({\theta }_{12},\phi ),$$

where *ρ*_*λ*∩_, _*rel*_ (*θ*_*12*_, *φ*) is relative spectral normal hemispherical reflectance and *ρ*_*λref*_ (*θ*_*12*_, *φ*) calibrated spectral reflectance of DHR standard.

The calibrated uncoated specular Molybdenum laser mirror (MoStan) with reflectance higher than 0.95 from the company Laser Beam Products Ltd, UK is used as the DHR standard. Specular and diffuse samples and materials with different degree of reflectance are measured with this standard. The long-term stability of MoStan can be affected by the surface oxidation. Therefore, a periodic standard calibration is performed.

Four sample spectral signals *V*_*λ*_ (*θ*_*12*_, *φ*_*i*_) are usually collected for four sample positions with azimuth angle 0°, 45°, 90° and 135°. The sample spectral signal *V*_*λ*_ (*θ*_*12*_, *φ*) is calculated as the average of the measured signals.

Reference and sample spectral signals are detected from the area of 8 mm in x-direction and 8 mm in y-direction (Fig. S3b and Analyzed Area Dimensions section online).

Reflectance evaluation of semitransparent coatings deposited on a transparent substrate is complicated by the fact, that the coating cannot be measured independently without substrate. While the spectral signal of the DHR standard includes only the radiation reflected from its surface, the measured spectral signal of the coating/substrate system depends on the reflectance of the first coating surface *ρ*_*λ*∩,*C1*_ (*θ*_*12*_, *φ*), the transmittance of the coating *τ*_*λ*∩,*C*_ (*θ*_*0*_, *φ*), the reflectance of the coating/substrate interface *ρ*_*λ*∩,*C2/SUB1*_ (*θ*_*12*_, *φ*), the transmittance of substrate *τ*_*λ*∩,*SUB*_ (*θ*_*0*_, *φ*), and the reflectance of the second surface of substrate *ρ*_*λ*∩,*SUB2*_ (*θ*_*12*_, *φ*).

**Figure 6:**
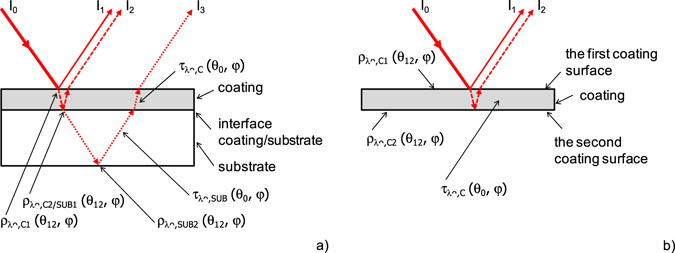
Schematic view of single-reflectance in sample. (**a**) Semitransparent coating deposited on transparent substrate. (**b**) Semitransparent coating without substrate.

When the single-reflectance of radiation is considered (Fig. 6a), the spectral signal of coating/substrate system *V*_*λ*_ (*θ*_*12*_, *φ*) is directly proportional to the sum of individual radiation intensities

4 $${V}_{\lambda }({\theta }_{12},\phi )=c\cdot ({I}_{1}+{I}_{2}+{I}_{3}),$$

where *c* is proportionality constant and the individual radiation intensities are expressed as follows

5 $${I}_{1}={I}_{0}\cdot {\rho }_{\lambda \cap ,C1}({\theta }_{12},\phi ),$$

6 $${I}_{2}={I}_{0}\cdot {\tau }_{\lambda \cap ,C}^{2}({\theta }_{0},\phi )\cdot {\rho }_{\lambda \cap ,C2/SUB1}({\theta }_{12},\phi ),$$

7 $${I}_{3}={I}_{0}\cdot {\tau }_{\lambda \cap ,C}^{2}({\theta }_{0},\phi )\cdot {\tau }_{\lambda \cap ,SUB}^{2}({\theta }_{0},\phi )\cdot {\rho }_{\lambda \cap ,SUB2}({\theta }_{12},\phi ),$$

where *I*_*0*_ is intensity of incident radiation. Similarly, the reference signal *V*_*λref*_ (*θ*_*12*_, *φ*) is formulated

8 $${V}_{\lambda ref}({\theta }_{12},\phi )=c\cdot {I}_{ref}=c\cdot {I}_{0}\cdot {\rho }_{\lambda ref}({\theta }_{12},\phi ).$$

The spectral normal hemispherical reflectance of coating/substrate system can be expressed by substituting equations (4–8) in the general formula (3)

9 $$\begin{array}{rcl}{\rho }_{\lambda \cap }({\theta }_{12},\phi ) & = & {\rho }_{\lambda \cap ,C1}({\theta }_{12},\phi )+{\tau }_{\lambda \cap ,C}^{2}({\theta }_{0},\phi )\cdot {\rho }_{\lambda \cap ,C2/SUB1}({\theta }_{12},\phi )\\  &  & +{\tau }_{\lambda \cap ,C}^{2}({\theta }_{0},\phi )\cdot {\tau }_{\lambda \cap ,SUB}^{2}({\theta }_{0},\phi )\cdot {\rho }_{\lambda \cap ,SUB2}({\theta }_{12},\phi ).\end{array}$$

The substrate transmittance *τ*_*λ*∩,*SUB*_ (*θ*_*0*_, *φ*) and coating transmittance *τ*_*λ*∩,*C*_ (*θ*_*0*_, *φ*) is determined by the procedure described in Transmittance Measurement section.

The edge reflectance of transparent substrate *ρ*_*λ*∩,*SUB2*_ (*θ*_*12*_, *φ*) can be evaluated from the measurement of substrate transmittance and reflectance according to

10 $${\rho }_{\lambda \cap ,SUB2}({\theta }_{12},\phi )=\frac{{\rho }_{\lambda \cap ,SUB}({\theta }_{12},\phi )}{1+{\tau }_{\lambda \cap ,SUB}^{2}({\theta }_{0},\phi )}.$$

The reflectance of the coating/substrate system *ρ*_*λ*∩_ (*θ*_*12*_, *φ*) is the measured value. The remaining unknown quantities in the equation (9) are the reflectance of the coating/substrate interface *ρ*_*λ*∩,*C2/SUB1*_ (*θ*_*12*_, *φ*) and the reflectance of the first coating surface *ρ*_*λ*∩,*C1*_ (*θ*_*12*_, *φ*). These two quantities can be calculated by the solution of two equations with two unknowns, when the reflectance and transmittance of two coating/substrate systems with different known thickness of the same coating are analyzed.

However, the reflectance of semitransparent coating itself *ρ*_*λ*∩,*C*_ (*θ*_*12*_, *φ*) is not identical with the reflectance of the first coating surface *ρ*_*λ*∩,*C1*_ (*θ*_*12*_, *φ*). When the single-reflectance of radiation is considered (Fig. 6b), the reflectance spectral signal of semitransparent coating itself *V*_*λ*,*C*_ (*θ*_*12*_, *φ*) is directly proportional to the sum of individual radiation intensities

11 $${V}_{\lambda ,C}({\theta }_{12},\phi )=c\cdot ({I}_{1}+{I}_{2}),$$

where *c* is proportionality constant and *I*_*1*_ and *I*_*2*_ are individual radiation intensities. Intensities *I*_*1*_ and *I*_*2*_ are expressed

12 $${I}_{1}={I}_{0}\cdot {\rho }_{\lambda \cap ,C1}({\theta }_{12},\phi ),$$

13 $${I}_{2}={I}_{0}\cdot {\tau }_{\lambda \cap ,C}^{2}({\theta }_{0},\phi )\cdot {\rho }_{\lambda \cap ,C2}({\theta }_{12},\phi ),$$

where *I*_*0*_ is intensity of incident radiation and *ρ*_*λ*∩,*C2*_ (*θ*_*12*_, *φ*) is reflectance of the second coating surface.

When the equations (8, 11–13) are substituted in equation (3), the spectral normal hemispherical reflectance of coating itself is calculated as

14 $${\rho }_{\lambda \cap ,C}({\theta }_{12},\phi )={\rho }_{\lambda \cap ,C1}({\theta }_{12},\phi )+{\tau }_{\lambda \cap ,C}^{2}({\theta }_{0},\phi )\cdot {\rho }_{\lambda \cap ,C2}({\theta }_{12},\phi ).$$

According to Fresnel’s equations^30^, it is valid that

15 $${\rho }_{\lambda \cap ,C1}({\theta }_{12},\phi )={\rho }_{\lambda \cap ,C2}({\theta }_{12},\phi ).$$

### Emissivity evaluation

The spectral normal emissivity of semitransparent coatings itself is calculated from the measured transmittance and reflectance according to Kirchhoff’s law^3^

16 $${\varepsilon }_{\lambda ,C}({\theta }_{0/12},\phi )=1-{\tau }_{\lambda \cap ,C}({\theta }_{0},\phi )-{\rho }_{\lambda \cap ,C}({\theta }_{12},\phi ).$$

The different angle of incidence on the analyzed sample in the transmittance and reflectance measurement is neglected. The emissivity is related to a specific thickness of analyzed coating.

Similarly, the emissivity of semitransparent bulk materials is evaluated according to

17 $${\varepsilon }_{\lambda }({\theta }_{0/12},\phi )=1-{\tau }_{\lambda \cap }({\theta }_{0},\phi )-{\rho }_{\lambda \cap }({\theta }_{12},\phi )$$

When the emissivity of opaque bulk materials is determined, the spectral normal transmittance is equal to 0 and the emissivity is computed according to

18 $${\varepsilon }_{\lambda }({\theta }_{12},\phi )=1-{\rho }_{\lambda \cap }({\theta }_{12},\phi )$$

### Measurement Uncertainty

The spectral normal emissivity of materials comprises an uncertainty. Generally, the emissivity uncertainty *μ* (*ε*_*λ*_) includes all particular uncertainties *μ* (*X*_*i*_) caused by variables in the equation for emissivity calculation. The total uncertainty is evaluated according to ref. 31 as a combined standard uncertainty of all particular uncertainties with coverage factor *k* = 2. All particular uncertainties are also established as the combined standard uncertainties of all particular sub-uncertainties *μ* (*Y*_*j*_).

The emissivity uncertainty of semitransparent coatings deposited on the transparent substrates *μ* (*ε*_*λ*,*C*_ (*θ*_*0/12*_, *φ*)) is evaluated by the equation

19 $$\mu ({\varepsilon }_{\lambda ,C}({\theta }_{0/12},\phi ))={[\sum _{i=1}^{n}{(\frac{\partial {\varepsilon }_{\lambda ,C}({\theta }_{0/12},\phi )}{\partial {X}_{i}}\cdot \mu ({X}_{i}))}^{2}]}^{\frac{1}{2}},$$

where *X*_*i*_ are individual sources of uncertainty from equation (16), *μ* (*X*_*i*_) the absolute particular uncertainties of individual sources, ${\rm{\partial }}{\varepsilon }_{\lambda ,C}({\theta }_{0/12},\phi )/{\rm{\partial }}{X}_{i}$ the respective sensitivity coefficients and $[({\rm{\partial }}{\varepsilon }_{\lambda ,C}({\theta }_{0/12},\phi )/{\rm{\partial }}{X}_{i})\cdot \mu ({X}_{i})]$ are the individual contributions of absolute particular uncertainty to total uncertainty of emissivity.

The absolute particular uncertainty of transmittance *μ* (*X*_*i*_) = *μ* (*τ*_*λ*∩,*C*_ (*θ*_*0*_, *φ*)) is evaluated as the combined standard uncertainty of all particular transmittance sub-uncertainties *μ* (*Y*_*j*_) = *μ* (*τ*_*i*_). The sources are shown in Table 1.

**Table 1:** Uncertainty sources of transmittance and reflectance measurement.

Individual source of uncertainty	Quantities
Repeatability of transmittance measurement	transmittance
Homogeneity of material for covering the reference port	transmittance
Interreflections involving the sample	transmittance
Repeatability of reflectance measurement	reflectance
DHR standard calibration	reflectance
DHR standard homogeneity	reflectance
(Inserting repeatability of anti-reflective window)	reflectance
(DHR standard reflectance with anti-reflective window)	reflectance
Inserting repeatability of integrating sphere system	transmittance, reflectance
Atmospheric spectral absorption	transmittance, reflectance

The absolute particular uncertainty of reflectance *μ* (*X*_*i*_) = *μ*(*ρ*_*λ*∩,*C1*_ (*θ*_*12*_, *φ*)) is established as the combined standard uncertainty of all particular sub-uncertainties *μ* (*Y*_*j*_) = *μ* (*ρ*_*i*_) caused by variables in equation (9). Each variable is loaded by the additional uncertainty that is computed as the combined standard uncertainty. Basic uncertainty sources of the measured quantities are identical and shown in Table 1. The other potential uncertainty sources as spectrometer and electronics without interreflections^32^, and sphere, sample and beam geometry and the interaction between them^4^ are included in the repeatability of transmittance and reflectance measurement or neglected.

The uncertainties and sub-uncertainties depend on the wavelength, and transmittance and reflectance values of the analyzed sample. Therefore, the uncertainty must be computed for the specific wavelength and specific sample.

The same methodology is used to evaluate the uncertainty of both semitransparent and opaque bulk materials.

## Experimental Results

**Figure 7:**
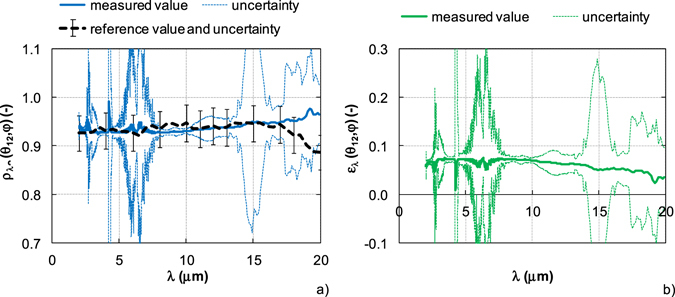
Results of opaque bulk diffuse material – AuStan. (**a**) Measured reflectance vs. reference value. (**b**) Evaluated emissivity.

**Figure 8:**
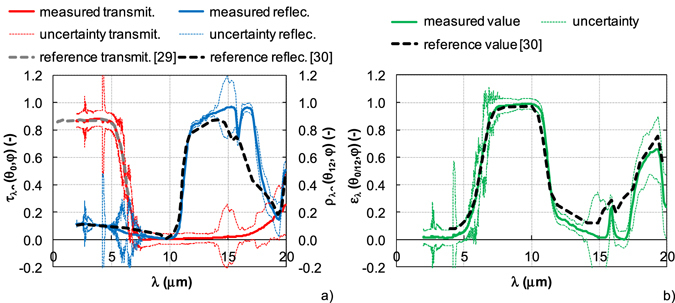
The results of semitransparent bulk material – sapphire (1 mm thickness). (**a**) Measured transmittance and reflectance vs. reference values. (**b**) Evaluated emissivity vs. reference value.

In order to validate the experimental device and measurement and evaluation procedures, a number of materials have been analyzed by the integrating sphere system. Several examples are shown in Figs 7 and 8. The results of transmittance, reflectance and emissivity are compared with the datasheet^33^, the calibration certificate and the values measured in other laboratories^34, 35^.

Avian Gold Diffuse Standard (AuStan) presents the opaque bulk diffuse material with high reflectance (about 0.93). The spectral normal hemispherical reflectance (measured value) was determined by equation (3) and the emissivity evaluated according to equation (18). The AuStan was certified by the Avian Technologies LLS, USA in 2013 for the spectral normal hemispherical reflectance in the spectral range from 2 μm to 20 μm and at a 7° incidence angle (reference value). The measured value is compared with the reference value in Fig. 7a, the evaluated emissivity is shown in Fig. 7b. The results include the measurement uncertainties.

The measured reflectance of AuStan (Fig. 7a) is about 0.93 in the whole spectral range. This slightly increases with increasing wavelength. In comparison to the reference value, the measured value is also the same in the spectral range from 2 μm to 17 μm. Above 17 μm, a difference is evident between the measured and reference value. The difference is 0.08 at the wavelength of 20 μm. If the uncertainties of reference and measured value are reflected, the measured reflectance AuStan corresponds to the reference value in the spectral range from 2 μm to 20 μm. The different incidence angle between the reference and measured value (7° for reference value and 12° for measured value) can be neglected. In both cases, the incidence angle is less than 20° to the surface normal^36^.

The uncertainty of reference value is indicated by the uncertainty bars and it is 0.04. The uncertainty of measured reflectance is less than 0.02 for the wavelengths up to 16 μm. In the spectral range from 16 μm to 20 μm the uncertainty is higher, approximately 0.1. Higher uncertainty for the long wavelengths is caused by the reduced spectral response of mercuric cadmium telluride (MCT) detector of integrating sphere system^37^. The reflectance uncertainty is mostly influenced by the uncertainty of atmospheric spectral absorption. All uncertainties are presented for the 95% confidence level (coverage factor *k* = 2).

The reflectance spectrum is affected by the change of atmospheric absorptions due to varying partial pressures of H_2_O and CO_2_ in spectral ranges 2.5–2.95 μm, 4.17–4.5 μm, 4.8–8 μm and 13.2–17.2 μm. In the atmospheric absorption bands the upper limit of uncertainty exceeds the value of one. The changes of atmospheric absorption affect the measured reflectance spectrum minimally. The real reflectance value is of course between the lower limit of uncertainty and one.

The evaluated emissivity of AuStan including uncertainty (coverage factor *k* = 2) is shown in Fig. 7b. The emissivity is less than 0.1 in whole spectral range. With increasing wavelength the emissivity slightly decreases. Analogous to the reflectance, the emissivity also comprises an uncertainty. The emissivity uncertainty is less than 0.02 for the wavelengths up to 16 μm. In the spectral range from 16 μm to 20 μm, the uncertainty varies about 0.1. The emissivity uncertainty involves both the reflectance uncertainty that is mainly caused by the uncertainty of atmospheric spectral absorption and the transmittance uncertainty mostly influenced by the interreflections involving the sample (Table 1). In the atmospheric absorption bands the lower limit exceeds the value of zero. The real emissivity value is between the upper limit of uncertainty and zero.

An example of transparent bulk specular material is shown in Fig. 8. The transmittance, reflectance and emissivity of an uncoated sapphire window with thickness of 1 mm from the company UQG Ltd., UK were analyzed including the uncertainty. The results are compared with the reference values^33–35^. The measured value is indicated by full line, the reference value by dashed line.

The spectral normal transmittance and reflectance of sapphire window are shown in Fig. 8a. The measured transmittance corresponds to the reference value^33^. In the spectral range from 2 μm to 5 μm, the transmittance is about 0.87, over 5 μm rapidly decreases and the window is opaque at the wavelengths above 7.5 μm. The reflectance shows the opposite trend. The reflectance of sapphire window is less than 0.2 at the wavelengths up to 10 μm. Above 10 μm, the reflectance rapidly increases and the values are higher than 0.8 in the spectral range from 12 μm to 17 μm (the reflectance is close to 1 at the wavelength 15 μm). Then, the reflectance decreases to 0.15 at the wavelength of 19.5 μm. The sample has no transmittance and reflectance at the wavelength about 10 μm (Christiansen wavelength)^38^. As in Fig. 7, the spectra are affected by the atmospheric absorption changes.

The measured reflectance is compared with the spectrum specified in^34^. The reference spectrum is measured under the incidence angle of 15° in the spectral range from 6.25 μm to 384 μm at ambient temperature (~25 °C). The reference is a sapphire with unknown thickness with two polished faces. The measured and reference reflectance curves are in good agreement, except in the spectral range from 11 μm to 19.5 μm. The reference reflectance is there lower than measured value. The differences can be caused by variations in the material itself and used measurement methods.

The transmittance uncertainty is higher than the reflectance uncertainty. The transmittance and reflectance uncertainty is less than 0.1 for the wavelengths up to 17 μm except the atmospheric absorption bands. While the reflectance uncertainty is less than 0.1 at the wavelengths over 17 μm, the transmittance uncertainty increases up to 0.2.

The spectral normal emissivity of sapphire window is calculated from the measured values according to equation (17). In Fig. 8b., the final emissivity curve is compared to an emissivity curve published in ref. 34 (reference value). The measured spectrum corresponds to the reference spectrum up to wavelength of 12 μm, the curves diverge over 12 μm. The difference is 0.3 at the wavelength of 17 μm. The differences can be due to variations in the material itself and used measurement methods. While the evaluated emissivity of sapphire window with the thickness of 1 mm is calculated from the transmittance and reflectance measured at ambient temperature under the incidence angle of 12°, the reference value is indicated for the sample with thickness of 0.79 mm at temperature of −70 °C and measured under the incidence angle of 0°.

The evaluated emissivity of sapphire window was also compared to data published in ref. 35. Sova *et al*. analyzed the emissivity of UV-grade sapphire with thickness of 1 mm over the temperature range from 480 °C to 1650 °C ± 50 °C. The results report that with increasing temperature, Christiansen wavelength (about 11 μm at the temperature 480 °C ± 50 °C) shifts to the higher values and the multiphoton absorption edge shifts to shorter wavelengths. Some differences are evident between the emissivity spectrum at ambient temperature (Fig. 8b) and the reference spectrum at the temperature of 480 °C ± 50 °C. However, the differences correspond to the different temperatures. Christiansen wavelength for the emissivity at ambient temperature is about 10 μm, at the temperature of 480 °C ± 50 °C is about 11 μm, i. e. Christiansen wavelength shifts to longer wavelengths with increasing temperature. Similarly, the multiphoton absorption edge is at longer wavelengths for emissivity spectrum at ambient temperature than at high temperature. The spectral emissivity spectrum at ambient temperature corresponds to the high temperature emissivity curve.

The experimental device and measurement and evaluation procedures have been validated by the measurement of optical properties of opaque bulk diffuse material and transparent bulk specular material. In the last example, the results of optical properties for semitransparent coating are presented.

**Figure 9:**
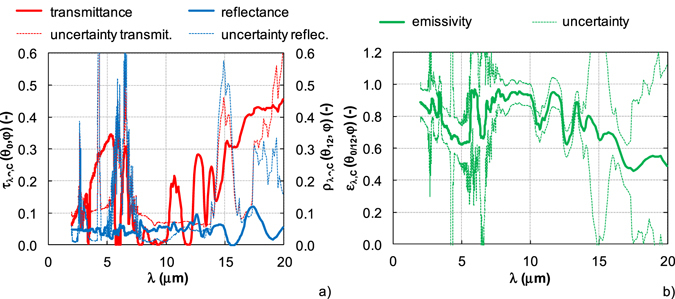
The results of semitransparent coating (23 μm thickness) deposited on transparent substrate – DupliColor Aerosol Art RAL 9500/Ge substrate. (**a**) Transmittance and reflectance. (**b**) Emissivity.

DupliColor Aerosol Art RAL 9500 coating (MOTIP DUPLI LTD, Germany) deposited on an germanium uncoated window with thickness of 1 mm from the company UQG Ltd., UK presents semitransparent coating deposited on a transparent substrate. Two samples with different coating thickness were prepared, the first one with thickness of 10 μm and the second one with thickness of 23 μm. The transmittance, reflectance and emissivity were evaluated and the results of optical properties are presented in Fig. 9 for the selected coating thickness (23 μm).

The spectral normal hemispherical transmittance and reflectance are shown in Fig. 9a. The coating transmittance reflects a distinct dependence on the wavelength. It is in the interval from 0 to 0.5. No transmittance is in the spectral ranges 5.7–6.1 μm, 7.6–8 μm, 9.1–9.8 μm and 11.8–12.2 μm, the maximum transmittance is at wavelength 20 μm. The reflectance is less than 0.1 in the whole spectral range. The reflectance uncertainty is lower than the transmittance uncertainty and in both cases the uncertainty increases with increasing wavelength. At 20 μm the transmittance uncertainty is 0.5 and the reflectance uncertainty is about 0.25. These high values of both uncertainties are the sum of individual sources of uncertainty according to equation (19). The transmittance uncertainty is most affected by the uncertainty of interreflections involving the sample, the uncertainty of atmospheric spectral absorption contributes most to the reflectance uncertainty.

The evaluated emissivity spectrum is shown in Fig. 9b. The emissivity varies with wavelength. At wavelengths from 2 μm to 5 μm the emissivity decreases from 0.88 to 0.63. In the spectral range from 5 μm to 12 μm the values are close to 1 and above 12 μm the emissivity gradually decreases to value of 0.5. The emissivity uncertainty is about 0.1 for the wavelengths up to 16 μm. In the spectral range from 16 μm to 20 μm the uncertainty increases, at 20 μm it is about 0.5. The emissivity uncertainty of 0.5 is sum of individual sources of transmittance and reflectance uncertainty.

This coating is used for high emissivity purposes at high temperatures. From the results it can be seen that it has high emissivity in the range from 5 μm to 12 μm, but not continuously and not outside of this region. This is important for practical applications. The transmissivity of the coating means that when applied to different substrates the resulting emissivity of coating/substrate system will be different. The benefit of the present method is that the transmissivity of the coating is measured and emissivity of the coating itself can be predicted for different coating thicknesses.

**Figure 10:**
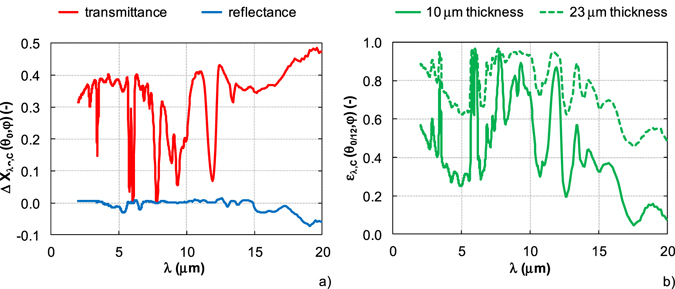
Differences of optical properties of semitransparent coating DupliColor Aerosol Art RAL 9500/Ge substrate between coating thickness of 10 μm and 23 μm. (**a**) Absolute difference of transmittance and reflectance. (**b**) Emissivity for two different coating thicknesses.

Figure 10a illustrates the absolute difference of transmittance and reflectance of DupliColor Aerosol Art RAL 9500 coating for two different coating thicknesses of 10 μm and 23 μm. For transmittance, it is evident that the absolute difference varies with wavelength. At wavelengths about 6 μm and 7.8 μm the coating is opaque for both analyzed thicknesses. Small differences bellow 0.1 are distinct for wavelengths 9.3 μm and 12 μm. The thinner coating shows higher transmittance of 0.3–0.4 than coating with higher thickness in wavelength band from 2 μm to 7.5 μm and for wavelengths over 12.5 μm. On the contrary, the different coating thickness does not influence the spectral reflectance up to 15 μm. Over 15 μm, the reflectance of coating with thickness of 23 μm is higher than the reflectance of coating with thickness of 10 μm. Thus, the variation of spectral emissivity of DupliColor Aerosol Art RAL 9500 coating for both analyzed thicknesses (Fig. 10b) is mainly influenced by different coating transmittance. The higher absolute values of emissivity are obtained for the coating with the higher thickness (23 μm) in the whole spectral range. The absorption bands of coating about 6 μm and 7.8 μm cause high coating emissivity independent of coating thickness.

## Conclusion

It was developed a new measurement method, which can be used for determination of spectral emissivity of coatings on transparent substrates at room temperature through reflectance and transmittance measurements. The method is significant by selection of appropriate and precisely calibrated reference standard for reflectance, in detail definition of measurement procedures and addition of accessories for transmittance measurement. The reference standard material was selected from five tested standards with opaque and specular surfaces and different levels of reflectance. Uncoated molybdenum laser mirror with reflectance higher than 0.95 (specular, calibrated in Laboratoire National d’Essais in France) was selected for the absolute reflectance measurement of diffuse and specular materials and materials with any degree of reflectance. Two transmittance accessories were designed for the bulk samples measurement (or coating on a substrate). They allow the measurement of various samples (shapes and sizes). The analyzed sample is inserted into the accessory sample port and locked into the measurement position by using the spacers and distance ring. For accurate transmittance measurement, the same distance ring position should be used (space filled by spacers). The detector of integrating sphere system must be cooled by liquid nitrogen after the connection to the interface of spectrometer and then stabilized for 140 minutes. The detector sensitivity is stable for 6.5 hours after cooling and stabilization. The analyzed area dimensions are 8 mm for the reflectance and from 19 mm to 22 mm for the transmittance measurement. The back reflected radiation of integrating sphere system is negligible and is not included to the reflectance and transmittance measurement. The measurement uncertainty was assessed.

The method was tested and verified on opaque (gold coated diffuse standard) and transparent bulk (sapphire) samples. The results show that the measured values correspond to the reference values (datasheet, calibration certificate and values measured in other laboratories) if the uncertainty is included. Finally, as an example of semitransparent coating deposited on a transparent substrate, there are presented results of DupliColor Aerosol Art RAL 9005 coating with thickness of 23 and 10 μm deposited on the germanium uncoated window with thickness of 1 mm. The reflectance is less than 0.1, the transmittance and the emissivity varies with the wavelength. For 23 μm thick film, there is no transmittance in the spectral ranges 5.7–6.1 μm, 7.6–8 μm, 9.1–9.8 μm and 11.8–12.2 μm, the maximum transmittance of 0.45 is at wavelength 20 μm. The emissivity is higher than 0.5, the average value is about 0.81. The transmittance and emissivity values vary significantly with coating thickness. The uncertainty increases with increasing wavelength. Except the mentioned atmospheric absorption bands the determined transmittance uncertainty is about 0.1 up to 16 μm, at 20 μm it is about 0.5. The reflectance uncertainty is half of the transmittance uncertainty; the emissivity uncertainty is similar to the transmittance uncertainty.

Development of this method enables a deep analysis of emissivity of semitransparent coatings at room temperature without necessity of heating of samples. This opens application potential to different fields like biomedical and nanotechnologies.

## Electronic supplementary material

Method for emissivity measurement of semitransparent coatings at ambient temperature
