# Supplementary material for: Method for emissivity measurement of semitransparent coatings at ambient temperature
Source: Sci Rep. 2017 May 3;7:1386. doi: 10.1038/s41598-017-01574-x (PMC5431223; doi:10.1038/s41598-017-01574-x)
Supplement: Supplementary file 1 — Method for emissivity measurement of semitransparent coatings at ambient temperature [file 41598_2017_1574_MOESM1_ESM.pdf]

Article title

Method for emissivity measurement of semitransparent coatings at ambient temperature

Authors and addresses

Petra Honnerová[a *]

e-mail: petrahon@ntc.zcu.cz

phone: +420 377 634 719

Jiří Martan[a]

e-mail: jmartan@ntc.zcu.cz

Zdeněk Veselý[a]

e-mail: zvesely@ntc.zcu.cz

Milan Honner[a]

e-mail: honner@ntc.zcu.cz

[a] New Technologies Research Centre (NTC), University of West Bohemia, Univerzitní 8, 306 14, Pilsen, Czech Republic

[*] Corresponding author

SUPPLEMENTARY INFORMATION

Integrating sphere system operation

Operation of integrating sphere system comprises:

- connection of integrating sphere system to interface of the spectrometer,
- temperature stabilization of integrating sphere system after connection to interface,
- time stability of integrating sphere system,
- and temperature stability of integrating sphere system.

The Hg:Cd:Te detector of integrating sphere system requires a cooling by liquid nitrogen. The detector can be cooled in two modes. In the first mode, the detector is cooled after the connection to the interface of spectrometer and sufficient stabilization of the detector is ensured before the measurement of spectral quantities. In the second mode, the cooling and stabilization of the detector is performed before the connection to the interface of spectrometer. The spectral quantities are measured immediately after the connection of detector to the interface. But it is not clear that the second mode allows the measurement of spectral signals immediately after the connection of detector to the interface or an additional stabilization of the detector is required. Therefore, the second mode was simulated. A set of spectral signals reflected from the standard reference material MoStan located on the reflectance sample port was measured in time (every 10 minutes from the connection of detector to the interface of spectrometer during detector cooling) and the differences between spectral signals $\Delta V_{\lambda\ \Delta t}\ (\theta_{12}, \varphi)$ were evaluated. Selected differences are shown in Fig. S1a.

If the detector is cooled after the connection to the interface of spectrometer, sufficient stabilization of the detector should be ensured before the measurement of spectral quantities. The time required for the stabilization was analyzed. The spectral signals reflected from the standard reference material MoStan placed on the reflectance sample port were measured every 10 minutes for 150 minutes from the detector cooling. The differences between the spectral signals $\Delta V_{\lambda\ \Delta t}\ (\theta_{12}, \varphi)$ were calculated and the selected are shown in Fig. S1b.

The reflectance and transmittance measurement require a reference spectral signal measurement (the reflected radiation from a standard reference material for reflectance measurement, transmitted radiation by the transmittance accessory without a sample or by the transmittance accessory with a transparent substrate), which is related to the sample signal. The time stability of integrating sphere system means the time period of possibility to use the same reference spectral signal without affecting the reflectance and transmittance results. The spectral signals reflected from the standard reference material MoStan positioned on the

reflectance sample port were measured every 10 minutes for 50 minutes after sufficient temperature stabilization of the integrating sphere system. The absolute values of differences between ith and the first reference signal $\Delta V_{\lambda\,\Delta t}\,(\theta_{12},\,\varphi)$ were calculated (Fig. S1c).

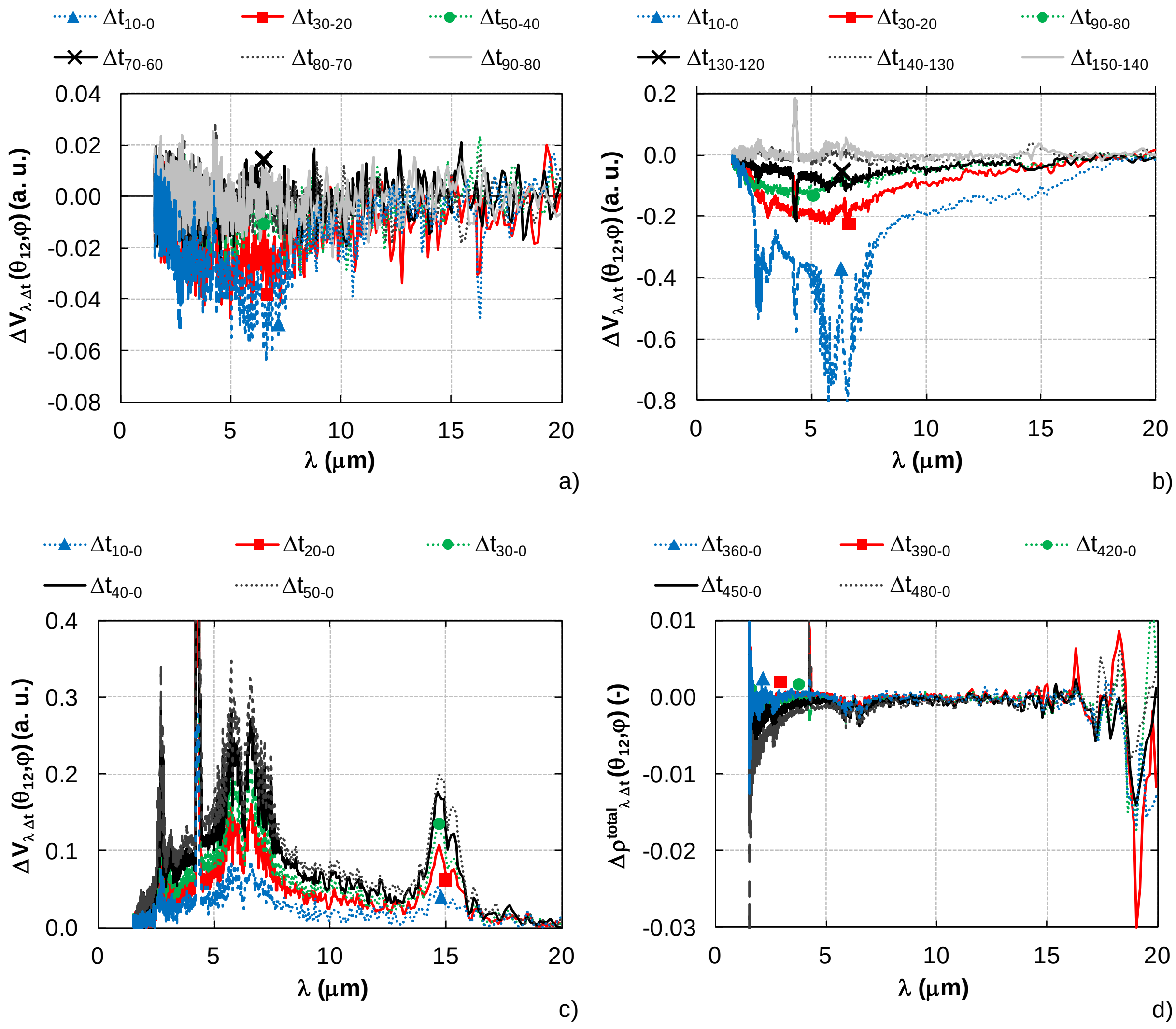

Figure S1. *Integrating sphere system operation. (a) Connection of integrating sphere system with temperature stabilized detector to interface of the spectrometer. (b) Detector temperature stabilization of integrating sphere system after connection to interface. (c) Time stability of integrating sphere system. (d) Detector temperature stability of integrating sphere system.*

The temperature stability of integrating sphere system defines the utilization time of integrating sphere system without changing the detector sensitivity after the cooling and the temperature stabilization. First, the integrating sphere system was stabilized for 2 hours. Subsequently, the reference and sample signal were measured for MoStan placed on the

reflectance sample port. The signals were measured every 30 minutes for 8 hours. Total line of reflectivity for MoStan was evaluated as the ratio of sample and reference signal at a given time. Finally, the differences between ith and the first total line of reflectivity $\Delta\rho^{total}{}_{\lambda\,\Delta t}\,(\theta_{12},\,\varphi)$ were calculated. In Fig. S1d, the results show the changes in the spectral sensitivity of integrating sphere system for the times longer than 360 minutes after the temperature stabilization.

The results of integrating sphere system operation can be summarized as follows.

- When the detector is cooled and stabilized before the connection of integrating sphere system to the interface of spectrometer, subsequent stabilization is required after the connection of detector to the spectrometer, see Fig. S1a. Therefore, it is advisable to connect the integrating sphere system and then cool and stabilize the detector.
- The time differences between the spectral signals tend to be negligible after 140 minutes from the connection of integrating sphere system to the interface of spectrometer and its subsequent cooling by liquid nitrogen (Fig. S1b). To ensure sufficient detector stability, it is required to observe this time before the spectral reflectance and transmittance measurement.
- The differences between the measured spectral signal at certain time and the spectral signal at initial time increases with increasing the time difference. The changes are observed already at the time difference of 10 minutes (Fig. S1c). Therefore, it is recommended to measure the reference spectral signal before the measurement of spectral signal for each sample.
- For short wavelengths, less than 5 μm, the differences of total line of reflectivity for MoStan occur after 7 hours from the connection of detector to the interface of spectrometer and 2 hours detector stabilization. For long wavelengths over 15 μm, the differences of total line of reflectivity for MoStan are evident from the start of measurement. These are not probably caused by a change in spectral sensitivity of the detector in time. Under this assumption, it is possible to conclude that the detector sensitivity (Fig. S1d) is stable at the periodic measurement of reference spectral signal for 6.5 hours after connection and stabilization of the detector. Then, it is necessary to cool and stabilize the detector again.

The results are affected by changing of atmospheric absorptions due to variation of partial pressures of H_2O and CO_2 in spectral ranges from 2.5 μm to 2.95 μm, from 4.17 μm to 4.5 μm, from 4.8 μm to 8 μm and from 13.2 μm to 17.2 μm.

Back reflected radiation

The reflectance and transmittance measurement can be affected by the back reflected radiation from the inner surface of integrating sphere system. If the back reflected radiation contributes significantly to the measured quantities, it has to be included in the evaluation.

The back reflected radiation from the inner surface of integrating sphere system was analyzed in the reflectance mode. Two spectral signals were measured. At first, the reference spectral signal was measured with MoStan positioned on the reflectance port. For the detection of the second signal (sample signal), no standard reference material or sample was used (the reflectance port of the sphere was empty). The sample signal corresponds only to the back reflected radiation from the inner surface of integrating sphere system. The back reflected radiation (Fig. S2) was calculated as a ratio of both signals (sample vs. reference).

The back reflected radiation of integrating sphere system is less than 0.01 over the entire spectral range. This value is negligible in comparison to the estimated measurement uncertainty. Therefore, it is not necessary to include the back reflected radiation to the reflectance and transmittance measurement

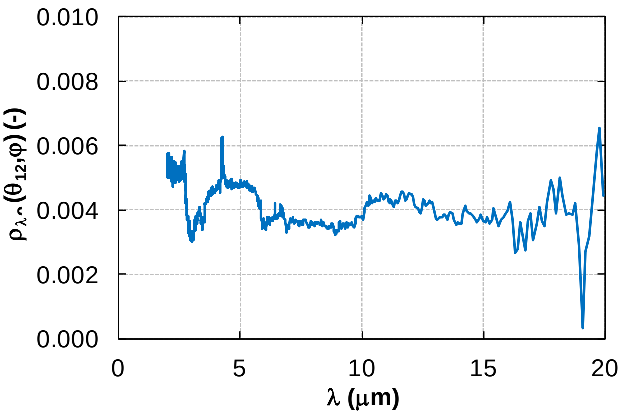

Figure S2. *Back reflected radiation of integrated sphere system in reflectance mode.*

Analyzed area dimensions

Analyzed area dimensions were evaluated for the reflectance and transmittance measurement. For transmittance measurement, the analyzed area dimensions depend on the sample thickness (0.01 to 7 mm). If the linear relationship between the analyzed area dimension and the sample thickness is considered, the analyzed area dimension is determined for each sample thickness according to the equation

$$analyzed\ area\ dimension = a \cdot L + b, \qquad (1)$$

where L is the sample thickness, the coefficients a and b are determined later.

The analyzed area dimension was determined by the aperture (a thin aluminum sheet of a thickness 0.01 mm with a circular hole of a specific size) which was inserted to the transmittance accessory to simulate the limit values of sample thickness of 0.01 mm and 7 mm. For the minimum sample thickness, the aperture was inserted to the accessory between the stop pad and the o-ring. In the second case, the aperture has been inserted between the spacer and the distance ring. The aperture diameters from 14 mm to 22 mm and from 18 mm to 22 mm with increments of 1 mm were used.

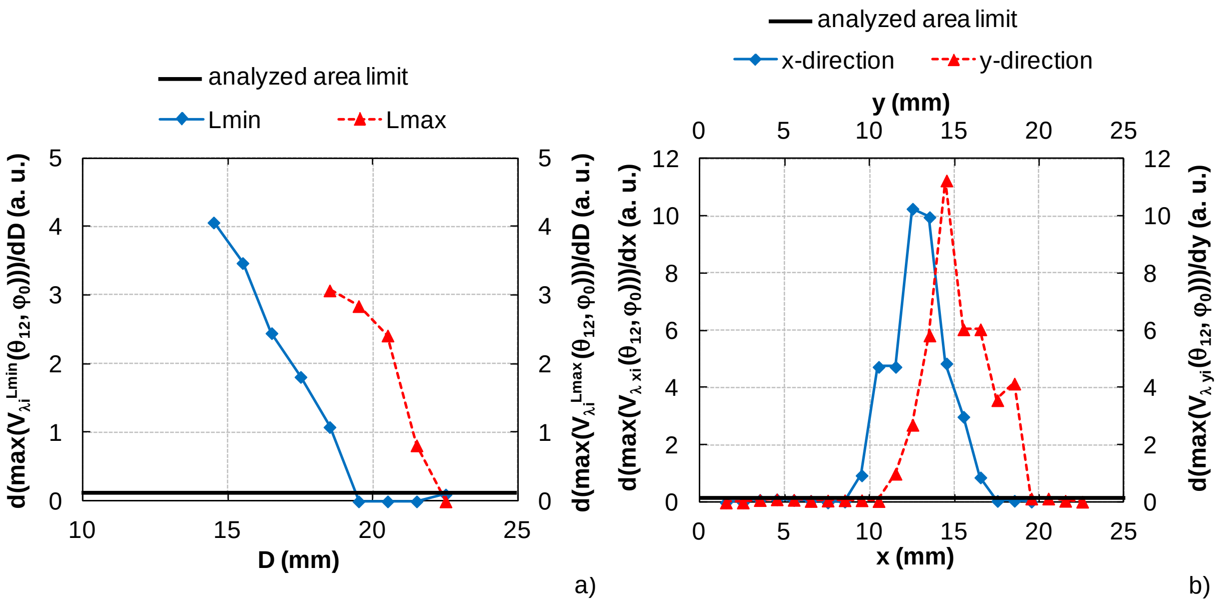

Figure S3. *Analyzed area dimensions. (a) Transmittance measurement. (b) Reflectance measurement.*

The spectral signal without aperture and several spectral signals with the apertures of different diameters and different positions were measured subsequently and the maximum values of spectral signals have been deducted. The derivation of maximum values of spectral signals according to the aperture diameter were calculated for the two aperture positions simulating the minimum sample thickness ($d(max(V_{\lambda i}^{Lmin}(\theta_{12}, \varphi_0)))/dD$) and the maximum sample thickness ($d(max(V_{\lambda i}^{Lmax}(\theta_{12}, \varphi_0)))/dD$). The results are shown in Fig. S3a. The derivations are higher than 0.1 for the case when the aperture limits the incident radiation. Therefore, the analyzed area dimension is in accordance with the aperture diameter for which a change of maximum values of spectral signals is higher than 0.1. Thus, the analyzed area dimension is defined for the limit sample thickness of 0.01 mm and 7 mm. A linear regression curve is used for the determination of coefficients *a* and *b* of equation (1).

The analyzed area dimension is determined to 19 mm for the samples with minimum thickness of 0.01. Similarly, the analyzed area dimension is 22 mm for the limit sample thickness of 7 mm (maximum). The analyzed area dimension has been calculated for each sample thickness according to equation (1) with the coefficients a = 0.4292 and b = 18.9960.

For the reflectance measurement, square sample with the edge size of 60 mm was used. Half of the sample was equipped with a low-emissivity aluminum film. A high-emissivity coating DupliColor 800 °C (MOTIP DUPLI Ltd, Germany) was deposited on the second half of the sample. Spectral signals were detected for the sample situated on the reflectance sample port of integrating sphere system in different positions. The initial sample position was selected so that the reflectance sample port was covered by the part of the sample with low-emissivity aluminum film. Other sample positions corresponded to a shift of 1 mm towards a part of the sample with high emissivity coating. The last detected signal corresponded to the reflectance from the high emissivity part of the sample only. In total, twenty spectral signals for the sample moved in the x-direction and twenty four spectral signals for the sample moved in the y-direction were measured. The maximum values of spectral signals were deducted and their derivations were evaluated for x-direction $d(max(V_{\lambda\,xi}\ (\theta_{12},\ \varphi_0)))/dx$ and y-direction $d(max(V_{\lambda\,yi}\ (\theta_{12},\ \varphi_0)))/dy$. The analyzed area dimension corresponds the area of $d(max(V_{\lambda\,xi}\ (\theta_{12},\ \varphi_0)))/dx > 0.1$ and $d(max(V_{\lambda\,yi}\ (\theta_{12},\ \varphi_0)))/dy > 0.1$ (Fig. S3b). The analyzed area dimensions are 8 mm in x-direction and 8 mm in y-direction.
